# Interactive effects of dietary protein and fiber levels on total tract and apparent ileal nutrient digestibility, microbiota profiling, and fermentation products in pigs fed a blend of branched-chain volatile fatty acids

**DOI:** 10.3389/fvets.2025.1731832

**Published:** 2026-01-26

**Authors:** Angie P. Benavides-Infante, Anlly M. Fresno-Rueda, Lucas Alves Rodrigues, Michael T. Socha, Tatiane Fernandes, Benoit St-Pierre, Jorge Y. Perez-Palencia, Crystal L. Levesque

**Affiliations:** 1 Department of Animal Science, South Dakota State University, Brookings, SD, United States; 2 Zinpro Corporation, Eden Prairie, MN, United States; 3 School of Animal Sciences, Virginia Polytechnic Institute and State University, Blacksburg, VA, United States

**Keywords:** digestibility, fiber, isobutyrate, isovalerate, microbiome, pigs, protein

## Abstract

**Introduction:**

The experiment investigated the interactions between diet protein and fiber and branched-chain volatile fatty acids (BCVFA) on nutrient digestion, fermentation products, and microbiome modulation in pigs.

**Methods:**

Fourteen cannulated pigs (body weight 20.4 ± 1.4 kg) were used in a replicated 6 × 5 Youden square design with 6 diets and 5 periods for at least 11 observations per dietary treatment. Experimental diets were 2 × 2 + 2 arrangement, consisting of 4 BCVFA-diets (isobutyrate, isovalerate, and 2-methyl butyrate, 1:1:1) supplemented at 1%, with varying protein [low (LP; 15%) or normal (NP; 19%) protein] and fiber [low (LF: 11%) neutral detergent fiber (NDF) or high (HF: 17% NDF)]. The ‘+2’ diets were a positive LP-LF (PC) and a negative NP-HF (NC) control without BCVFA. Diets, fecal, and ileal digesta samples were analyzed for nutrients, fiber composition, AA, and titanium. Fecal samples were analyzed for bacterial composition.

**Results:**

Pigs fed BCVFA-supplemented NP-HF diets had the greatest AID for acid detergent fiber (ADF), acid detergent lignin, cellulose, and hemicellulose, and the greatest ATTD for ADF (*p* < 0.05). The AID of AA was greater (*p* < 0.05) in pigs fed LP-LF PC diet compared to LP-LF BCVFA-supplemented diet (*p* < 0.05). There were no interactions (*p* > 0.05) between fiber and protein levels for volatile fatty acids (VFA) concentration in ileal and fecal samples. Feeding high fiber diets supplemented with BCVFA resulted in greater (*p* < 0.05) concentration of acetic, propionic, butyric, and total VFA production in fecal samples. Fecal bacteria affiliated to *Erysipelotrichaceae* were found in higher abundance in the BCVFA-supplemented NP-HF diet compared to its non-supplemented control (*p* < 0.05). Similarly, candidate bacterial strains of *Turicibacter sanguinis* (OTU Ssd-110) and *Romboutsia timonensi* (OTU Ssd-23) were more highly represented in the fecal microbial communities of pigs fed the BCVFA-supplemented NP-HF diet compared to its non-supplemented control (*p* < 0.05).

**Conclusion:**

Supplementation of 1% BCVFA in swine diets containing higher fiber and typical crude protein can optimize digestive efficiency, particularly at the ileal level, which was associated with improvements in nutrient digestibility potentially mediated by microbiome modulation. This may represent an opportunity to feed simpler diets, improving the efficiency and sustainability of swine production.

## 1 Introduction

In 2024, the world population was 8.2 billion people, with an expectation of continued growth reaching a population of 10.3 billion by 2080 (1). Consequently, a simultaneous increase in annual cereal and meat production will be required to meet the growing demand for food. As food demand for humans rises, livestock animals that consume grains, such as corn, wheat, and soybeans, will become prospective competitors to the human food supply and security (2). In this context, feeding strategies that incorporate alternative feedstuffs for livestock, including co-products from agro-industrial crops, can reduce competition for human food ingredients and contribute to sustainable meat animal production. In addition, incorporating alternative ingredients in livestock diets can reduce feed costs and increase profitability (3). However, these alternative ingredients typically contain high fiber levels in the form of structural carbohydrates (non-starch polysaccharides, NSP) and antinutritional factors (ANFs) that affect nutrient digestibility, thereby compromising animal growth performance (4, 5).

The inclusion of high fiber ingredients in swine diets is usually limited, particularly in growing pig diets, due to the negative effect of fiber components on dietary nutrient utilization (3). High fiber diets have lower nutritional value for pigs because host enzymes secreted in the small intestine cannot degrade NSP (6). Also related to its physical–chemical characteristics, such as viscosity, diets with high fiber content have been attributed with reduced nutrient digestibility (7); when the digesta viscosity increases and digestive efficiency is negatively impacted, growth performance (i.e., final body weight) and intestinal morphology have been reported to be impaired (8). In this context, feed additives that promote fiber degradation, and consequently improve the nutritional value of high fiber diets for pigs, represent an opportunity to improve efficiency and sustainability of swine production by better using available co-products from agro-industrial crops that are currently considered waste (9–11).

Currently, different feed additives such as enzymes, probiotics, prebiotics, and acidifiers have been used to improve digestibility or reduce the negative impacts generated by antinutritional factors of feed ingredients (12, 13). This is one of the reasons why new strategies continue to emerge, as is the case with the branched-chain volatile fatty acids (BCVFA) isobutyrate, isovalerate, and 2-methyl butyrate which have been investigated as promising feed additives to improve nutrient utilization in livestock (14–16). Most research to date on BCVFA supplementation has been conducted on ruminants, specifically cattle, and it has shown to improve digestibility and nutrient absorption. However, it is necessary to consider that ruminants and pigs differ in the structure of their digestive system. In ruminants, microbial fermentation primarily takes place in the rumen, the largest of their four stomach compartments, and it precedes gastric digestion; while microbial fermentation also occurs in the ruminant hindgut, its contribution to host nutrition is not as critical compared to the rumen. In contrast, microbial fermentation in pigs occurs mainly in the hindgut, more specifically the large intestine and the cecum (17). These structural differences influence the location of some nutrient absorption, the potential for fiber fermentation to contribute substantially to whole body nutrient supply and, consequently, the microbial composition (18). Considering the aforementioned differences, this raises the question of whether any improvement can be observed in pigs by supplementing their diets with BCVFA.

Dietary BCVFA inclusion in ruminant diets has been shown to improve fiber digestibility (19–21) by promoting the proliferation of fiber-digesting organisms in the rumen (22, 23), resulting in improved milk fat, milk yield, and fiber degradability (including neutral detergent fiber digestibility), as well as production of VFAs such as acetate (24, 25). In swine, a previously published study by our group has indicated that inclusion of a BCVFA blend (isobutyrate, isovalerate, and 2-methyl butyrate, 1:1:1) at 1% in a normal protein, low fiber diet fed to growing pigs can increase digestibility. This was observed at the total tract level for gross energy (GE), crude protein (CP), and hemicellulose, as well as for essential amino acids (Ile, Leu, Phe, and Thr) in the ileal compartment (26). However, since it is a product that has only recently been researched in pigs, there is limited information available about the mechanism of action through which BCVFA can promote nutrient utilization. In addition, their impact on fiber digestion has not been established in pigs. As symbiotic gut microbial communities may be involved in this process, the potential modulation of the gut microbiome in response to BCVFA supplementation also needs to be further explored.

In this context, the study described in this report aimed to investigate the independent and interactive effects of dietary protein and fiber levels on apparent ileal (AID) and total tract nutrient digestibility (ATTD), microbiota profiling, as well as fermentation products in BCVFA-fed pigs. Together, the results from these experiments will allow a better understanding of the interactions between diet composition and BCVFA supplementation, including the identification of key gut microbial taxa regulated by BCVFA in swine and their associations with nutrient digestibility.

## 2 Materials and methods

### 2.1 Animal and housing

The protocol for this project was approved by the Institutional Animal Care and Use Committee at South Dakota State University (No. 2104-020A). Fourteen crossbred barrows with an initial body weight of 20.4 ± 1.4 kg were selected and surgically modified with a T-cannula at the distal ileum. One week before surgery, pigs were moved from group to individual pens for adaptation to individual housing. After surgery, pigs were housed in individual crates for post-surgical care and recovery. At d7 post-surgery, pigs were moved to individual floor pens in a temperature-controlled room for the entire experimental period. All pens contained one dry self-feeder, as well as one nipple waterer for ad libitum access to water.

### 2.2 Experimental diets

Experimental diets (Table 1) were organized in a 2 × 2 + 2 treatment arrangement, consisting of BCVFA-diets supplemented at 1%, with varying protein [low (LP: 15%) or normal (NP: 19%) protein] and fiber levels [low (LF: 2.5%) crude fiber (CF), 11% neutral detergent fiber (NDF) or high (HF: 5.0% CF, 17% NDF) fiber]. In addition, two control diets non-supplemented (without BCVFA) were included: LP-LF (PC) and NP-HF (NC); based on their fiber inclusion levels, these were designated as positive and negative controls, respectively. The inclusion of the BCVFA blend was based on a previous experiment (26) (1% of an BCVFA blend: isobutyrate, isovalerate, and 2-methyl butyrate – 1:1:1). Diets were fortified with vitamins and minerals to meet or exceed the nutrient requirements of pigs (27). Titanium oxide (0.3%) was included in the diets as an indigestible marker to determine nutrient digestibility (28).

**Table 1:** Experimental diet formulation and calculated composition (as-fed basis)^1^.

Ingredient, %	NP-LF	NP-HF	LP-LF	LP-HF
Corn	60.85	43.25	79.41	60.27
Soybean meal	30.00	22.50	9.00	2.00
Corn distillers dried grains with solubles	3.00	16.50	3.00	17.00
Sugar beet pulp	2.00	11.00	2.00	11.50
Resistant starch^2^	0.00	0.50	0.00	0.50
L-Lysine HCl, 98.5%	0.06	0.23	0.73	0.88
DL-Methionine, 99%	0.01	0.01	0.18	0.18
L- Threonine, 98.5%	0.02	0.05	0.27	0.29
L-Tryptophan, 98%	0.00	0.02	0.11	0.14
L-Valine, 96.5%	0.00	0.03	0.35	0.36
L-Histidine, 98.5%	0.00	0.02	0.19	0.21
L-Phenylalanine, 98%	0.00	0.05	0.38	0.42
L-Isoleucine, 90%	0.00	0.05	0.39	0.43
L-Arginine, 98.5%	0.00	0.14	0.57	0.69
Soybean oil	1.42	3.34	0.42	2.48
Monocalcium phosphate	0.74	0.45	1.00	0.71
Limestone	1.14	1.12	1.20	1.18
Salt	0.13	0.11	0.17	0.13
Swine vitamin premix^3^	0.05	0.05	0.05	0.05
Swine mineral premix^4^	0.15	0.15	0.15	0.15
Titanium oxide	0.30	0.30	0.30	0.30
Swine larvicide	0.13	0.13	0.13	0.13
Nutrients in mixed feed				
ME, Kcal/kg	3,320	3,320	3,320	3,320
Crude protein, %	20.26	20.78	15.08	15.14
Crude fiber, %	2.76	5.02	2.48	4.86
NDF, %	11.47	16.70	11.11	16.59
ADF, %	4.83	8.12	3.91	7.39
SID Lys, %	0.98	0.98	0.98	0.98
SID Arg, %	1.11	1.11	1.11	1.11
SID Met, %	0.29	0.29	0.36	0.37
SID, Met + Cys, %	0.55	0.55	0.55	0.55
SID Thr, %	0.59	0.59	0.59	0.59
SID Trp, %	0.21	0.21	0.21	0.21
SID Ileu, %	0.78	0.78	0.78	0.78
SID Leu, %	1.52	1.55	1.03	1.08
SID Val, %	0.80	0.80	0.80	0.80
SID His, %	0.47	0.47	0.47	0.47
SID Phe, %	0.86	0.86	0.86	0.86
Calcium, %	0.66	0.66	0.66	0.66
Phosphorus, %	0.55	0.53	0.50	0.49
ATTD P, %	0.27	0.27	0.27	0.27
STTD P, %	0.31	0.31	0.31	0.31
Sodium, %	0.10	0.10	0.10	0.10
Ca/P	2.12	2.13	2.17	2.17

^1^A branched-chain volatile fatty acids blend (isobutyrate, isovalerate, and 2-methyl butyrate – 1:1:1) was supplemented and mixed on top of all the diets (1% of the diet). Two additional experimental diets were used: PC (LP-LF) and NC (NP-HF) without BCVFA supplementation. NP, normal protein; LP, low protein; LF, low fiber; HF, high fiber; NDF, neutral detergent fiber; ADF, acid detergent fiber; ME, metabolizable energy; SID, standard ileal digestibility; ATTD, apparent total tract digestibility; STTD, standardized total tract digestibility; Ca, Calcium; P, phosphorus.

^2^ MSP resistant starch. Box 850–10 Fredrick Street Carberry, Manitoba, Canada, R0K 0H0.

^3^J & R Distributing Inc. 518 Main Ave, Lake Norden, SD 57248 - USA. Minimum provided per kg of diet: vitamin A 11,023 IU, vitamin D3 1,653 IU, vitamin E 55 IU; vitamin B12 0.04 mg, menadione 4.41 mg, riboflavin 9.92 mg, d-pantothenic acid 60.63 mg, thiamine 3.31 mg, niacin 55.11 mg, vitamin B6 3.31 mg, folic acid 1.10 mg, biotin 0.17 mg.

^4^J & R Distributing Inc. 518 Main Ave, Lake Norden, SD 57248 - USA. Minimum provided per kg of diet: copper 16.5 mg, manganese 44.1 mg, selenium 0.3 mg, zinc 165 mg.

### 2.3 Experimental design and procedure

**Figure 1:**
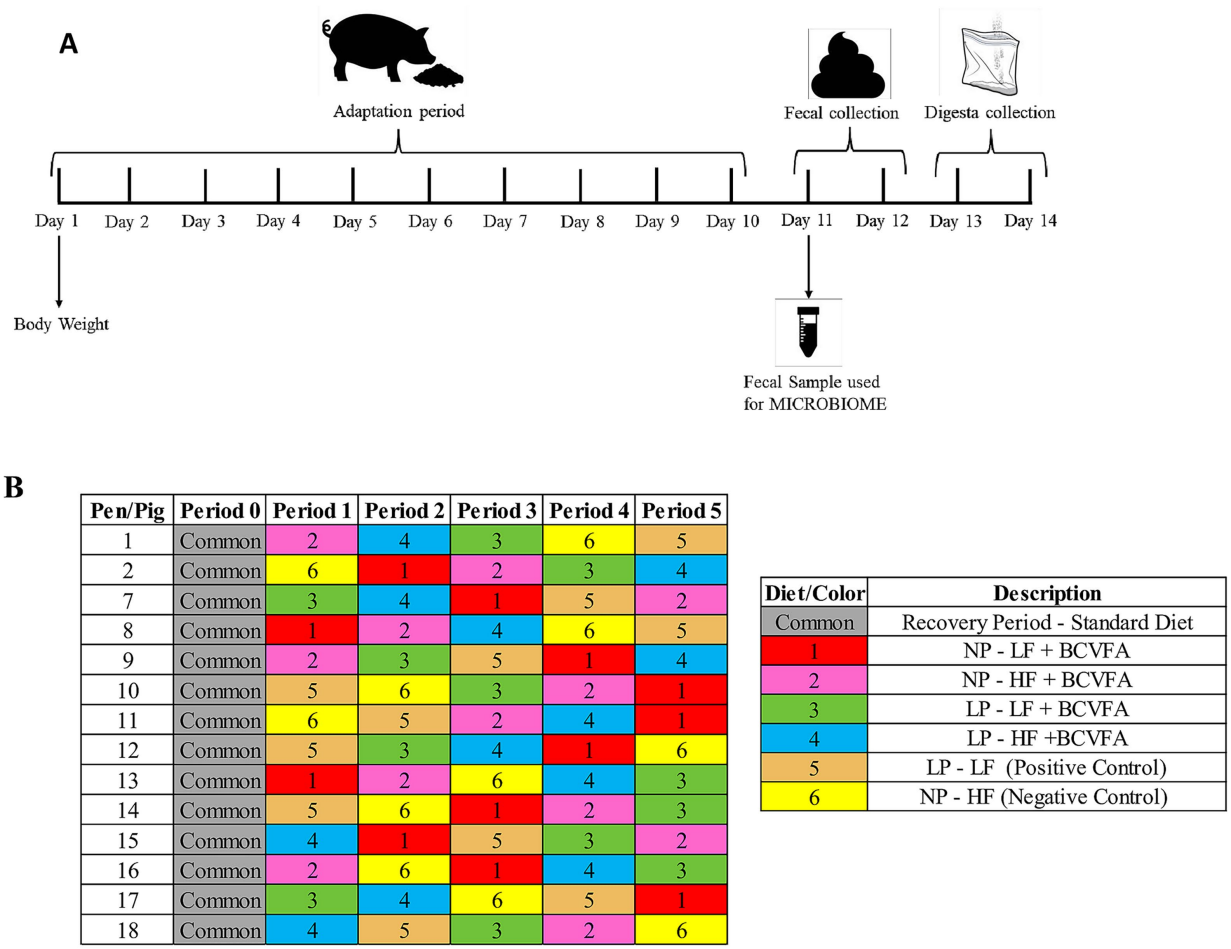
Experimental procedure for collecting fecal and digesta samples in a single collection period 14 days **(A)**. This procedure was repeated for 5 consecutive periods with each pig receiving a different diet in each period based on a random assignment for a total of 11 replications per diet **(B)**.

Pigs were distributed according to a replicated 6 × 5 Youden square design with 6 diets, 5 periods, and 2 or 3 replicate pigs per diet in each period; this design provided at least 11 observations for each dietary treatment (Figure 1). Throughout the experiment, daily animal care included monitoring pig behavior, recording daily room temperature (highest and lowest temperatures), feeding, checking waterers and feeders, as well as cleaning the cannula area. Zinc oxide was used on the skin around the cannula to minimize irritation from cannula leakage. Feed offered was set at 4% of body weight, which was adjusted according to individual pig weight at the beginning of each period (26). Feed was provided daily in two equal meals at 07:00 and 15:00 h, respectively. Each experimental period consisted of 14 d, including the first 10 d for adaptation to the diet, the next 2 d for feces collection, and the remaining 2 d for ileal digesta collection. At least 3 of adaptation is sufficient to stabilize indigestible marker for the purpose of determining nutrient digestibility in pigs (29) while there were minor changes in fecal microbiota (composition, diversity) after 10 days in diets containing different levels and types of dietary fibers (30). Consequently, an adaptation period of 10 days is more than enough to ensure that the digesta from the previous diet has passed through the gastrointestinal tract before starting the next collection and to properly represent the associated change in microbiome. Pens were monitored three times throughout the day to collect freshly voided feces for each pig in separate sealable plastic bags (Ziploc®, SCJohnson, Racine, WI, USA), these samples were used for proximal analysis, GE, AA, fiber components, and titanium. For microbiome and ammonia analysis, a fecal sample (approximately 50 mL) was collected from each pig by rectal palpation on the first day of fecal collections once in the morning; these individual fecal samples were stored at −20 °C in separate 50 mL conical tubes until they were processed for DNA extraction. Ileal digesta was collected between 08:00 and 20:00 h into plastic bags with a capacity of 500 mL; these bags were removed when filled to approximately 70% capacity with digesta (or at minimum every 60 min), then the digesta were transferred to a larger capacity Ziploc bag labeled with the date and the experimental diet to be immediately frozen at −20 °C. Two separate sets of ileal digesta samples were collected in an alternative pattern during these periods: one sample was mixed with 5 mL of 10% (v/v) formic acid to minimize bacterial fermentation, while the following sample was not supplemented with formic acid, and was processed later for analysis of short-chain fatty acid (SCFA) content. At the end of each collection period, feces and digesta were, respectively, pooled by pig observation, homogenized, subsampled, then stored at −20 °C.

### 2.4 Sample preparation and chemical analysis

The digesta samples were freeze-dried (lyophilized) and fecal samples were dried in the oven at 102 °C. The lyophilized digesta and dried feces samples were ground to pass through a 0.5 mm screen using a mill grinder (Retsch zm 200, ring sieve size: 0.75 mm) before chemical analysis. The dry matter (DM), CP, amino acids (AA), ether extract (EE), ash, and titanium concentrations of the diets, digesta, and feces were determined. The DM concentration of feces was determined during the drying process and organic matter (OM) was calculated as a difference between DM and ash. Titanium concentration of the digesta and fecal samples was determined by spectrophotometry (model SpectraMAX190, Molecular Devices, Sunnyvale, CA, USA) at 408 nm after ashing at 525 °C for 10 h and digesting with anhydrous sodium sulfate and sulfuric acid at 120 °C for 24 h, following the procedure reported by Myers et al. (31). Neutral detergent fiber was analyzed using the Van Soest fiber fractionation system (JAOAC 56, 1,352–1,356, 1973), acid detergent fiber (ADF) and lignin (acid detergent lignin, ADL) followed AOAC Official Method 973.18 (acid detergent and H_2_SO_4,_ respectively) in animal feed. Hemicellulose was calculated as a difference between ADF and ADL, and cellulose was calculated as a difference between NDF and ADF. Crude fat and crude fiber were also analyzed following the AOAC Official Method 920.39 (Ether Extraction), Method 954.02, 2006 (Acid Hydrolysis) and Method 978.10, 2006, respectively. At a commercial laboratory (University of Missouri, Columbia MO), CP and AA concentration were analyzed using Official Method 990.03; AOAC International, 2007 and method 982.30 E; AOAC, 2005. Tryptophan concentration was analyzed by alkaline hydrolysis (AOAC Official Method 988.15, chp. 45.4.04, 2006) and Met and Cys, by performic acid oxidation/acid hydrolysis (AOAC Official Method 982.30 E(b), chp. 45.3.05, 2006). The GE of samples was measured using bomb calorimeter (Parr 6,300 calorimeter, Parr Instruments Co., Moline, IL, USA) according to the methods reported by Bech et al. (32). Briefly, 1 g of ground sample was pressed into a pellet using a pellet press and placed into the bomb calorimeter. The sample was analyzed in duplicate and repeated if the difference between the two values was more than 5%.

### 2.5 Short-chain fatty acids

Short chain fatty acids and lactic acids, referred to in combination as SCFA, were analyzed from ileal digesta and fecal samples by gas chromatography. All samples were processed according to Darwin et al. (33). Briefly, samples were thawed, and 2 ± 0.1 g samples were taken, diluted with 4 mL distilled water, vortexed for 3 min and let it rest overnight (4 °C). Then samples were centrifuged at 4,000 × g for 5 min and the supernatant filtered through a 0.22 μm pore size filter (Millex-GP), 0.57 mL of upper layer was mixed with 0.15 mL of internal standard (5 mmol/L, 4-methyl-valeric acid, 277,827, Sigma, St. Louis, MO, USA), acidified with 0.48 mL of periodic acid (100 mmol/L) and 0.3 mL of formic acid (10% w/v), and incubated at 100 °C for 1 h. The mixed solution was collected and used for SCFA determination using a 6,890 N Network GC System gas chromatograph (Agilent Technologies, Santa Clara, CA, USA) equipped with a flame ionization detector, according to Izuddin et al. (34). One microliter of the sample was injected at split 1:30, at a temperature of 230 °C. Separation of VFA profile was determined using Quadrex 007–10 Series (Quadrex Corp., New Haven, CT 06525, USA) bonded phase fused silica capillary column (15 m, 0.250 mm internal diameter, 0.25 μm film thickness). The temperature of the column was set at 60 °C and held for 2 min; increased to 100 °C (10 °C/min), increased to 200 °C (20 °C/min), and held for 2 min. Nitrogen gas was supplied as carrier gas at the rate of 1 mL/min. The temperature of the detector was set at 230 °C. Commercial standards (Sigma-Aldrich, St. Louis, MO) of lactic (1356734), acetic (45997), propionic (94425), iso-butyric (46935), butyric (19215), isovaleric (78651), valeric (75054), and caproic (21529) acids were used as external standards for gravimetrically prepare a calibration curve and peak identification. The molar concentration of SCFA was identified based on a single point of internal standard and calibration curve with external standards.

Ammonia was analyzed in ileal digesta and fecal samples using the method described by Novamsky et al. (35). This method involved the conversion of both ammonia and organic nitrogen-based compounds to ammonia. These compounds were combined with hypochlorite to form mono-chloramine. Further, the mono-chloramine reacted with salicylate and nitroprusside to produce a blue-colored compound called indosalicylate. The intensity of the resulting color was proportional to the concentration of ammonia. The samples were prepared by mixing them with water. Before analysis, the water portion of the samples was separated through centrifugation. A 1 mL aliquot of the supernatant was used for the analysis; this was diluted with 9 mL of deionized water (DI), and then 5.0 mL of the sample was transferred to the TNTplus Vial Test, HR (2–47 mg/L NH₃-N). The analysis was conducted for ammonia-N using the DR3900 spectrophotometer, and the results were multiplied by the dilution factor.

### 2.6 Microbial DNA isolation, PCR amplification of the 16S rRNA gene and amplicon sequencing

A 16S rRNA gene-based profiling approach was selected because it is a more comprehensive method for determining the bacterial composition of highly diverse microbial environments, such as the swine hindgut. Indeed, the swine gut microbiome includes a very high number of uncultured bacterial species whose respective genomes still remain to be elucidated; thus, while metagenomics would allow to determine the metabolic capabilities at the community level, assigning these functions to specific microbial species would lack in accuracy. Microbial genomic DNA was isolated from individual fecal samples (*n* = 83) using a repeated bead-beating plus column method, which included the QIAamp DNA Mini Kit (Qiagen, Hilden, Germany) (36). The V1-V3 region of the bacterial 16S rRNA gene was amplified by PCR using the universal forward 27F 5′AGAGTTTGATCMTGCTCAG and reverse 519R 5′GWATTACCGCGCGCGCTG primers (37). Purified microbial genomic DNA samples were provided to Molecular Research DNA (MRDNA, Shallowater, TX, USA) for V1-V3 PCR amplification, followed by amplicon sequencing with the Illumina MiSeq 2 × 300 platform to generate overlapping paired-end reads.

### 2.7 Computational analysis of PCR-generated 16S rRNA amplicon sequences

Raw sequence reads were quality filtered by selecting for (1) the presence of intact 27F and 519R primer nucleotide sequences, (2) a length of 400–580 bp, and (3) a minimal average Phred quality score of Q33. Quality-filtered sequences were first aligned, then clustered into operational taxonomic units (OTUs), using 4% sequence dissimilarity as a genetic distance cutoff; this threshold was selected instead of the more commonly used value of 3%, because the V1-V3 region is more variable than regions such as V3-V4, V4, or V4-V5 that are typically targeted for 16S rRNA gene-based composition analyses; this approach is based on previous reports (58, 59) showing that the V1-V3 region is more variable in comparison to the full length 16S rRNA gene [for more detailed procedures and justifications, please consult the approach described by Opdahl et al. (38)]. Next, 16S rRNA gene sequence artifacts were identified using the ‘chimera.uchime’ and ‘chimera.slayer’ commands from the MOTHUR open source software package (39), as well as by using an in-house database alignment search-based approach. After removal of sequence artifacts, the publicly available tools Ribosomal Database Project (RDP) Classifier (40) and BLAST (41) were used for taxonomic assignment of valid OTUs.

### 2.8 Calculations and statistical analysis

Apparent ileal digestibility (AID) values were calculated from the difference between the dietary intake of the nutrient and the concentration of the nutrient in the digesta present in the distal ileum of pigs according to the equation (42, 43):


$$\mathrm{AID},\%=100-[100\times (\frac{{\mathrm{Ti}}_{d}\times N_{i}}{{\mathrm{Ti}}_{i}\times N_{d}})]$$

Where Ti_d_ = concentration of titanium in the diet; Ti_i_ = concentration of titanium in ileal sample; N_i_ = concentration of the nutrient in ileal sample and N_d_ = concentration of the nutrient in the diet.

To determine the apparent total tract digestibility (ATTD) of nutrients, the following equation described by Adeola (44) was used:


$$\text{ATTD},\%=100-[\frac{\left({\mathrm{Ti}}_{d}\times N_{f}\right)}{{\mathrm{Ti}}_{f}\times N_{d}}]\times 100$$

Where digestibility refers to ATTD; Ti_d_ and Ti_f_ represent the concentrations of marker compounds in the diet and feces, respectively; and N_d_ and N_f_ represent the concentrations of nutrients in the feed and feces, respectively.

The UNIVARIATE procedure of SAS was used to confirm the homogeneity of variance and to analyze for outliers. Data was analyzed as a 2 × 2 factorial design using the PROC MIXED procedure in SAS. The model tested the main effects of dietary protein and dietary fiber and their interactions, considering a pig as the experimental unit and period the random effect. Tukey’s adjusted means test was performed to detect differences where *p* ≤ 0.05 was considered significant and 0.05 > *p* ≤ 0.10 a tendency. In addition, pre-planned contrasts were used to compare the diet LP-LF BCVFA supplemented vs. LP-LF non-supplemented and BCVFA NP-HF supplemented vs. NP-HF non-supplemented diet.

Statistical analysis of bacterial abundance among experimental diets was performed using the Kruskal–Wallis sum-rank test, as well as the pairwise Wilcoxon sum-rank test to compare abundances between pairs of treatment groups; the Wilcoxon sum-rank test also included the Benjamini–Hochberg correction to control the false discovery rate.

## 3 Results

Table 2 presents the analyzed composition of the experimental diets used in this experiment. Low-protein diets contained approximately 13% CP, and normal-protein diets contained 20% CP. Regarding fiber, low-fiber diets had 11% NDF, while high-fiber diets had approximately 16% NDF.

**Table 2:** Analyzed composition of experimental diets (as-fed basis)^1^.

Item	% BCVFA inclusion^1^				LP-LF^2^	NP-HF^3^
	NP-LF	NP-HF	LP-LF	LP-HF		
Crude protein, %	20.87	19.48	13.00	13.98	13.26	19.48
Dry matter, %	87.59	88.27	86.67	87.74	86.54	88.42
GE, Kcal/kg	3,925	4,157	3,788	3,933	3,781	4,081
Crude fat, %	2.23	3.65	2.06	3.02	2.55	5.15
Crude fiber, %	4.37	6.52	3.13	5.31	3.43	6.13
Ash, %	5.40	5.87	4.50	5.26	3.87	5.67
NDF, %	10.66	14.07	8.34	12.30	8.92	14.34
ADF, %	5.08	7.21	4.00	5.22	3.02	5.95
Cellulose, %	5.58	6.86	4.34	6.08	4.90	7.39
ADL, %	0.70	1.26	0.90	1.47	0.87	1.12
Hemicellulose, %	4.38	5.95	3.10	4.75	3.15	5.83
Total AA, %						
Aspartic Acid	1.91	1.82	0.87	0.83	0.90	1.89
Threonine	0.74	0.80	0.57	0.70	0.60	0.81
S erine	0.80	0.79	0.43	0.47	0.41	0.78
Glutamic Acid	3.48	3.36	1.86	1.91	1.91	3.48
Proline	1.13	1.24	0.72	0.86	0.74	1.25
Glycine	0.81	0.83	0.41	0.46	0.44	0.84
Alanine	0.97	1.07	0.61	0.73	0.64	1.08
Cysteine	0.32	0.34	0.19	0.21	0.20	0.37
Valine	0.98	1.03	0.78	0.89	0.82	1.10
Methionine	0.30	0.33	0.33	0.36	0.33	0.36
Isoleucine	0.86	0.89	0.70	0.81	0.73	0.95
Leucine	1.63	1.77	1.00	1.16	1.03	1.79
Tyrosine	0.64	0.66	0.35	0.41	0.36	0.66
Phenylalanine	0.95	1.01	0.88	0.91	0.81	1.04
Lysine	1.15	1.27	0.92	1.13	0.99	1.28
Histidine	0.52	0.55	0.39	0.47	0.44	0.57
Arginine	1.23	1.27	0.98	1.15	1.02	1.33
Tryptophan	0.24	0.22	0.20	0.22	0.20	0.23

^1^A BCVFA blend (isobutyrate, isovalerate, and 2-methyl butyrate – 1:1:1) was supplemented at 1% of the diet. BCVFA, branched-chain volatile fatty acids; NP, normal protein; LP, low protein; LF, low fiber; HF, high fiber; GE, gross energy; NDF, neutral detergent fiber; ADF, acid detergent fiber; ADL, acid detergent lignin; AA, amino acid.

^2^Positive control diet without BCVFA supplementation.

^3^Negative control diet without BCVFA supplementation.

### 3.1 Nutrient digestibility

There was an interaction (*p* < 0.05) between fiber and protein levels for the AID of DM, OM, GE, crude fat, ADF, ADL, and the AA Tau (Table 3). The LP-LF diet had greater (*p* < 0.05) AID of DM, OM, GE, and Tau compared to NP-LF and NP-HF diets. Diet with NP-HF content had a greater (*p* < 0.1) AID of crude fat, ADF, ADL, cellulose and hemicellulose compared to the other dietary treatments. There was no interaction between protein and fiber levels on the AID of CP and any of the essential AA. The AID of CP (*p* < 0.05) and most AA (*p* < 0.1) tended to be greater in NP diets compared to the LP diets. At the same time, HF diets tended to have reduced AID of CP (*p* < 0.1) and increased (*p* < 0.05) AID of Leu, Lys, and Met compared to LF diets. According to the contrast analysis, BCVFA improved (*p* < 0.05) AID of crude fiber, ADF, ADL, and hemicellulose in NP-HF diets (Table 4). As for LP-LF diets, AID of most nutrients decreased when BCVFA were supplemented compared to non-supplemented diet.

**Table 3:** Interactive and main effects of protein and fiber levels on apparent ileal digestibility of nutrients in growing pigs fed BCVFA-supplemented diets^1^.

Item	NP		LP		SEM	*p*-value^2^		
	LF	HF	LF	HF		Protein	Fiber	Protein × Fiber
CP	74.18	72.64	67.38	64.56	1.228	0.001	0.092	0.611
DM	67.81^b^	65.42^c^	74.34^a^	67.11^bc^	0.535	0.001	0.001	0.001
OM	72.08^b^	69.60^c^	78.82^a^	72.59^b^	0.456	0.001	0.001	0.001
GE	68.21^c^	68.97^bc^	78.38^a^	70.57^b^	0.611	0.001	0.001	0.001
Crude fat	66.85^b^	81.00^a^	68.07^b^	71.15^b^	1.254	0.001	0.001	0.001
Crude fiber	24.68	35.19	18.44	22.70	2.169	0.001	0.002	0.157
Ash	25.99	28.35	23.82	28.21	1.556	0.301	0.333	0.220
NDF	39.84	40.89	35.15	37.04	2.212	0.060	0.515	0.850
ADF	36.19^b^	44.98^a^	38.29^b^	38.98^ab^	1.646	0.240	0.007	0.018
Cellulose	46.79^y^	50.01^x^	50.09^x^	46.22^y^	1.867	0.897	0.863	0.064
ADL	61.57^b^	72.87^a^	72.15^a^	72.05^a^	2.146	0.030	0.015	0.012
Hemicellulose	31.92^y^	41.94^x^	28.27^y^	28.85^y^	2.419	0.001	0.036	0.057
Indispensable AA								
Arg	87.00	87.73	84.82	86.19	0.649	0.007	0.124	0.634
His	82.38	82.04	81.25	80.35	0.717	0.055	0.400	0.701
Ile	80.46	81.09	81.91	82.97	0.549	0.005	0.143	0.696
Leu	81.51	83.73	77.98	79.80	0.658	0.001	0.004	0.766
Lys	80.62	82.27	81.21	82.90	0.677	0.371	0.019	0.977
Met	85.03	86.59	88.20	88.56	0.477	0.001	0.056	0.224
Met + Cys	77.09	77.35	72.73	70.75	0.833	0.001	0.322	0.192
Phe	81.48	82.90	85.30	85.15	0.529	0.001	0.249	0.151
Thr	71.03	72.34	69.87	70.95	0.884	0.161	0.194	0.899
Trp	81.64	81.07	82.03	82.52	0.879	0.300	0.968	0.548
Val	77.18	77.37	77.43	77.12	0.693	0.996	0.928	0.723
Mean	81.15	81.69	81.02	81.62	0.582	0.870	0.343	0.957
Dispensable AA								
Ala	73.61	77.16	67.81	69.76	1.081	0.001	0.016	0.461
Asp	76.47^x^	75.90^x^	65.51^y^	61.24^z^	0.967	0.001	0.018	0.062
Cys	67.55^x^	68.23^x^	57.17^y^	52.88^y^	1.463	0.001	0.228	0.096
Glu	82.26	82.66	78.53	77.32	0.963	0.001	0.678	0.408
Gly	59.38	61.22	36.22	32.22	3.192	0.001	0.730	0.346
Ser	76.39	75.73	65.63	63.20	1.011	0.001	0.139	0.386
Tyr	80.16	80.97	72.53	72.87	0.777	0.001	0.467	0.764
Pro	61.98	62.22	45.87	41.73	5.464	0.003	0.737	0.704
Tau	67.91^c^	66.26^c^	81.70^a^	71.98^b^	1.036	0.001	0.001	0.001
Mean	71.76	72.28	63.60	60.55	1.307	0.001	0.351	0.185
Total AA mean	76.94	78.07	73.23	73.18	1.073	0.001	0.628	0.590

^1^A BCVFA blend (isobutyrate, isovalerate, and 2-methyl butyrate – 1:1:1) was supplemented at 1% of the diet. BCVFA, branched-chain volatile fatty acids; LF, low fiber; HF, high fiber. NP, normal protein; LP, low protein.

^2^Superscripts ^abc^ indicate significant difference at *p* ≤ 0.05 and ^xyz^ indicate tendency at 0.05 < *p* ≤ 0.10 using Tukey’s means separation test.

**Table 4:** Effects of dietary BCVFA supplementation on apparent ileal digestibility of nutrients in growing pigs fed LP-LF and NP-HF diets.

Item	LP-LF		NP-HF		SEM	*p*-value^1^	
	BCVFA^2^	Control	BCVFA^2^	Control		LP-LF Supp	NP-HF Supp
CP	67.36	72.44	72.64	72.64	1.101	0.002	0.830
DM	74.29	76.77	65.45	65.45	0.501	0.001	0.337
OM	78.78	80.87	69.62	69.62	0.438	0.002	0.303
GE	78.40	78.28	68.86	68.86	0.627	0.896	0.875
Crude fat	68.09	73.49	81.03	81.03	1.171	0.002	0.001
Crude fiber	18.32	23.75	35.10	35.10	2.799	0.080	0.008
Ash	23.76	23.97	28.38	28.38	1.519	0.924	0.365
NDF	35.03	41.65	40.91	40.91	1.929	0.021	0.532
ADF	38.03	43.53	45.16	45.16	1.593	0.020	0.004
Cellulose	49.96	59.77	50.09	50.09	1.638	0.001	0.381
ADL	71.94	72.55	73.14	73.14	2.059	0.837	0.026
Hemicellulose	28.05	35.57	42.07	42.07	2.165	0.019	0.005
Indispensable AA							
Arg	84.83	87.71	87.73	88.51	0.563	0.001	0.342
His	81.26	84.85	82.05	83.04	0.649	0.001	0.295
Ile	81.90	84.53	81.10	82.34	0.513	0.001	0.099
Leu	77.99	81.04	83.72	84.20	0.607	0.001	0.591
Lys	81.20	84.65	82.28	82.72	0.601	0.001	0.620
Met	88.19	89.82	86.60	87.63	0.455	0.016	0.125
Met + Cys	72.74	77.20	77.37	78.87	0.755	0.001	0.174
Phe	85.28	85.72	82.92	83.72	0.492	0.543	0.262
Thr	69.86	74.83	72.40	72.51	0.806	0.001	0.922
Trp	82.04	85.00	81.06	81.03	0.791	0.012	0.975
Val	77.42	81.02	77.39	78.99	0.628	0.001	0.084
Mean	81.01	83.90	81.71	82.47	0.534	0.001	0.328
Dispensable AA							
Ala	67.79	73.45	77.17	77.33	0.970	0.001	0.907
Asp	65.51	71.01	75.91	77.21	0.856	0.001	0.296
Cys	57.22	64.68	68.22	70.10	1.306	0.001	0.323
Glu	78.54	81.76	82.63	84.14	0.859	0.012	0.228
Gly	36.23	49.99	61.20	61.02	2.676	0.001	0.964
Ser	65.65	68.27	75.76	75.66	0.914	0.052	0.943
Tyr	72.54	76.42	80.97	80.64	0.720	0.001	0.753
Pro	45.87	56.75	61.78	60.75	5.145	0.167	0.890
Tau	81.56	81.02	66.37	65.09	0.974	0.698	0.365
Mean	63.64	69.27	72.23	72.45	1.208	0.002	0.904
Total AA mean	73.23	78.04	78.06	78.78	0.956	0.001	0.602

^1^Contrast: BCVFA supplemented diet vs. not supplemented diet.

^2^A BCVFA blend (isobutyrate, isovalerate, and 2-methyl butyrate – 1:1:1) was supplemented at 1% of the diet. BCVFA, branched-chain volatile fatty acids; NP, normal protein; LP, low protein; LF, low fiber; HF, high fiber.

There were interactions (Tables 5, *p* < 0.05) between fiber and protein levels for the ATTD of DM, OM, and GE, as well as for some dispensable AA (Leu, Met, Met + Cys, Phe), and most of the dispensable AA (except Pro, and Tau). The diet with LP-LF content had greater (*p* < 0.05) ATTD of DM, OM, and GE than the NP-HF diets. The ATTD of Met, Met + Cys, and Phe were greater in LP-LF than in LP-HF diets. Where an interaction was not detected, ATTD of CP, NDF, cellulose, and most AA was greater in NP and LF diets. Supplementation of BCVFA tended to improve ADF and ADL (*p* < 0.1) ATTD in LP-LF diet with no major impact on AA digestion (Table 6). In NP-HF diets, ATTD of DM, OM, GE, cellulose, and some AA (Arg, Ile, Met, Met + Cys, Val, Cys, and Tau) was greater (*p* < 0.05) in the control diet.

**Table 5:** Interactive effects of protein and fiber levels on apparent total tract digestibility of nutrients in growing pigs fed BCVFA-supplemented diets^1^.

Item	NP		LP		SEM	*p*-value^2^		
	LF	HF	LF	HF		Protein	Fiber	Protein × Fiber
CP	85.38	81.08	81.47	75.47	0.555	0.001	0.001	0.132
DM	83.35^b^	80.25^c^	85.51^a^	80.26^c^	0.428	0.015	0.001	0.016
OM	87.46^b^	84.15^c^	89.52^a^	84.45^c^	0.347	0.002	0.001	0.015
GE	85.22^b^	82.99^c^	87.16^a^	82.20^c^	0.371	0.132	0.001	0.001
Crude fat	54.36	62.76	61.78	62.43	3.690	0.347	0.236	0.304
Crude fiber	73.17	74.99	72.62	71.09	1.006	0.032	0.883	0.103
Ash	41.24	47.24	37.98	42.73	1.758	0.032	0.004	0.724
NDF	60.72	56.98	56.66	49.89	1.422	0.001	0.001	0.293
ADF	61.04	62.63	61.81	58.98	1.898	0.452	0.747	0.250
Cellulose	77.53	74.79	77.69	74.02	0.928	0.741	0.002	0.612
ADL	42.23^y^	48.07^y^	64.62^x^	55.61^xy^	4.279	0.002	0.726	0.097
Hemicellulose	65.83	65.69	61.15	59.82	1.527	0.001	0.636	0.701
Indispensable AA								
Arg	93.97	92.97	93.37	92.25	0.249	0.011	0.001	0.810
His	92.55	90.73	90.97	88.60	0.313	0.001	0.001	0.387
Ile	83.69	81.60	85.72	82.35	0.537	0.013	0.001	0.241
Leu	86.47^a^	85.63^ab^	84.06^b^	80.57^c^	0.525	0.001	0.001	0.015
Lys	87.20	85.81	88.09	85.53	0.540	0.574	0.001	0.287
Met	82.27^b^	79.90^c^	87.51^a^	82.63^b^	0.607	0.001	0.001	0.045
Met + Cys	84.19^a^	82.35^a^	83.68^a^	79.13^b^	0.531	0.001	0.001	0.014
Phe	86.14^b^	84.64^b^	89.10^a^	85.07^b^	0.448	0.001	0.001	0.007
Thr	82.48	80.33	82.95	79.62	0.539	0.823	0.001	0.275
Trp	93.73	91.46	94.52	92.34	0.331	0.016	0.001	0.898
Val	83.45^x^	81.18^y^	84.72^x^	80.44^y^	0.579	0.647	0.001	0.090
Mean	87.20	85.43	88.10	84.94	0.438	0.634	0.001	0.120
Dispensable AA								
Ala	78.88^x^	77.64^x^	76.45^x^	71.52^y^	0.934	0.001	0.002	0.055
Asp	87.64^a^	84.20^b^	79.62^c^	68.81^d^	0.649	0.001	0.001	0.001
Cys	86.11^a^	84.8^a^	79.85^b^	75.63^c^	0.565	0.001	0.001	0.014
Glu	91.15^a^	89.27^b^	86.91^c^	82.46^d^	0.458	0.001	0.001	0.007
Gly	81.38^a^	78.37 ^b^	72.94^c^	65.19^d^	0.765	0.001	0.001	0.004
Ser	88.10^a^	85.79^b^	82.72^c^	77.62^d^	0.444	0.001	0.001	0.003
Tyr	86.20^a^	83.72^b^	81.26^c^	76.31^d^	0.592	0.001	0.001	0.043
Pro	90.08	89.18	86.58	84.37	0.404	0.001	0.001	0.110
Tau	94.41	91.57	95.98	92.99	0.238	0.001	0.001	0.752
Mean	87.10^a^	84.95^b^	82.48^c^	77.21^d^	0.504	0.001	0.001	0.004
Total AA mean	87.35^a^	85.35^b^	85.45^b^	81.35^c^	0.481	0.001	0.001	0.034

^1^A BCVFA blend (isobutyrate, isovalerate, and 2-methyl butyrate – 1:1:1) was supplemented at 1% of the diet. BCVFA, branched-chain volatile fatty acids; NP, normal protein; LP, low protein; LF, low fiber; HF, high fiber. NP, normal protein; LP, low protein.

^2^Superscripts ^abcd^ indicate significant difference at *p* ≤ 0.05 and ^xy^ indicate tendency at 0.05 < *p* ≤ 0.10 using Tukey’s means separation test.

**Table 6:** Effects of dietary BCVFA supplementation on apparent total tract digestibility of nutrients in growing pigs fed LP-LF and NP-HF diets.

Item	LP-LF		NP-HF		SEM	*p*-value^1^	
	BCVFA^2^	Control	BCVFA^2^	Control		LP-LF Supp	NP-HF Supp
CP	81.46	82.08	81.06	81.29	0.540	0.424	0.763
DM	85.48	85.40	80.26	81.90	0.421	0.892	0.009
OM	89.50	89.08	84.15	85.55	0.344	0.396	0.007
GE	87.14	86.64	82.98	86.78	0.362	0.337	0.001
Crude fat	61.62	63.09	62.78	72.23	3.381	0.765	0.058
Crude fiber	72.58	70.07	75.02	75.53	1.066	0.108	0.744
Ash	37.96	37.99	47.24	50.80	1.792	0.990	0.175
NDF	56.50	53.86	57.11	61.58	1.546	0.242	0.059
ADF	61.70	56.81	62.80	59.87	2.003	0.097	0.316
Cellulose	77.63	77.24	74.84	79.22	0.948	0.779	0.002
ADL	64.40	51.45	48.49	26.07	4.906	0.007	0.003
Hemicellulose	61.04	58.27	65.78	66.67	1.591	0.233	0.700
Indispensable AA							
Arg	93.36	93.78	92.97	93.73	0.250	0.258	0.039
His	90.96	92.01	90.73	91.60	0.312	0.023	0.057
Ile	85.70	86.62	81.57	83.34	0.505	0.216	0.019
Leu	84.05	84.80	85.60	86.36	0.500	0.302	0.302
Lys	88.08	88.75	85.78	86.36	0.494	0.348	0.422
Met	87.49	87.73	79.88	81.93	0.564	0.762	0.015
Met + Cys	83.67	84.96	82.31	84.29	0.502	0.082	0.009
Phe	89.08	88.37	84.63	85.69	0.428	0.257	0.091
Thr	82.93	83.50	80.32	81.32	0.512	0.440	0.185
Trp	94.52	94.48	91.43	92.12	0.307	0.933	0.125
Val	84.70	85.80	81.15	82.80	0.533	0.160	0.037
Mean	88.09	88.58	85.41	86.53	0.414	0.409	0.066
Dispensable AA							
Ala	76.41	77.62	77.60	78.59	0.867	0.338	0.431
Asp	79.59	80.35	84.18	85.07	0.583	0.371	0.293
Cys	79.86	82.18	84.75	86.64	0.537	0.004	0.018
Glu	86.90	87.54	89.25	90.01	0.427	0.304	0.218
Gly	72.91	74.91	78.33	79.06	0.704	0.054	0.478
Ser	82.69	81.56	85.77	86.60	0.457	0.092	0.217
Tyr	81.21	81.66	83.70	83.22	0.571	0.589	0.557
Pro	86.57	87.05	89.17	89.71	0.394	0.406	0.343
Tau	95.98	95.58	91.57	92.39	0.247	0.273	0.026
Mean	83.46	83.16	84.92	85.70	0.472	0.308	0.261
Total AA mean	85.44	86.00	85.33	86.22	0.450	0.390	0.177

^1^Contrast: BCVFA supplemented diet vs. not supplemented diet.

^2^A BCVFA blend (isobutyrate, isovalerate, and 2-methyl butyrate – 1:1:1) was supplemented at 1% of the diet. BCVFA, branched-chain volatile fatty acids; NP, normal protein; LP, low protein; LF, low fiber; HF, high fiber.

### 3.2 Fermentation products

There were no interactions (*p* > 0.05) between fiber and protein levels for VFA concentration in ileal and fecal samples (Tables 7, 8). Feeding low protein diets supplemented with BCVFA resulted in lower (*p* < 0.05) concentration of ammonia on ileal digesta samples and lower (*p* = 0.075) proportion of acetic acid in fecal samples compared to the normal protein diets supplemented with BCVFA. Feeding high fiber diets supplemented with BCVFA resulted in lower (*p* < 0.05) concentration of lactic acid on ileal digesta samples and higher (*p* < 0.05) concentration of acetic, propionic, butyric, and total VFA production in fecal samples compared to the low fiber diets supplemented with BCVFA.

**Table 7:** Interactive effects of protein and fiber levels on ileal volatile fatty acids of growing pigs fed BCVFA-supplemented diets^1^.

Item	NP		LP		LP-LF^2^	NP-HF^2^	SEM	*p–*value^3^			
	LF	HF	LF	HF				Protein	Fiber	LP-LF Supp^4^	NP-HF Supp^4^
Ammonia, mg/L	329.40	344.69	258.58	237.16	251.12	316.64	13.615	0.001	0.829	0.635	0.108
VFA, mmol/L^5^											
Lactic	0.69	0.47	0.80	0.53	0.88	0.47	0.064	0.675	0.001	0.044	0.943
Acetic	16.50	14.74	17.37	18.04	21.58	12.88	1.347	0.134	0.695	0.034	0.346
Propionic	3.74	3.62	3.85	4.03	4.15	3.49	0.404	0.523	0.946	0.592	0.794
iso-Butyric	0.10	0.10	0.12	0.20	0.09	0.00	0.040	0.136	0.341	0.571	0.079
Butyric	1.90	1.25	1.83	1.76	2.69	1.33	0.238	0.350	0.142	0.017	0.795
iso-Valeric	0.24	0.24	0.32	0.30	0.25	0.24	0.060	0.226	0.822	0.389	0.993
Valeric	0.09	0.15	0.11	0.16	0.17	0.17	0.073	0.810	0.462	0.601	0.803
Caproic	0.12	0.04	0.01	0.07	0.03	0.00	0.049	0.245	0.889	0.656	0.489
Total VFA	22.70	20.12	23.68	20.12	28.80	20.54	1.702	0.123	0.631	0.079	0.824
VFA, % of total^6^											
Acetic	72.94	71.45	74.44	73.53	74.27	71.20	1.920	0.355	0.540	0.950	0.958
Propionic	17.14	19.95	16.47	16.45	15.02	18.26	1.924	0.283	0.475	0.595	0.514
iso-Butyric	0.35	0.43	0.46	0.80	0.30	0.02	0.162	0.154	0.210	0.434	0.058
Butyric	7.72	6.03	7.10	7.18	9.01	6.70	0.563	0.637	0.166	0.029	0.423
iso-Valeric	1.00	1.19	1.18	1.19	0.77	0.92	0.242	0.714	0.699	0.238	0.419
Valeric	0.35	0.79	0.33	0.60	0.53	0.88	0.316	0.752	0.271	0.676	0.828
Caproic	0.50	0.17	0.03	0.24	0.08	0.01	0.195	0.314	0.758	0.826	0.454

^1^A BCVFA blend (isobutyrate, isovalerate, and 2-methyl butyrate – 1:1:1) was supplemented at 1% of the diet. BCVFA, branched-chain volatile fatty acids; NP, normal protein; LP, low protein; LF, low fiber; HF, high fiber; VFA, volatile fatty acid. NP, normal protein; LP, low protein.

^2^Two additional diets without BCVFA were included (LP-LF and NP-HF).

^3^There was no interaction (*p* > 0.05) between fiber and protein levels on the ileal VFA concentration.

^4^Contrast: BCVFA supplemented diet vs. not supplemented diet.

^5^VFA, mmol/L: average millimoles of VFAs are present per liter of sample.

^6^VFA, % of total: Average of the percentage distribution of each VFAs within the samples of each diet.

**Table 8:** Interactive effects of protein and fiber levels on fecal volatile fatty acids of growing pigs fed BCVFA-supplemented diets^1^.

Item	NP		LP		LP-LF^2^	NP-HF^2^	SEM	*p*–value^3^			
	LF	HF	LF	HF				Protein	Fiber	LP-LF Supp^4^	NP-HF Supp^4^
Ammonia, mg/L	465.91	481.21	404.91	467.68	405.61	486.17	39.397	0.356	0.337	0.984	0.968
VFA, mmol/L^5^											
Lactic	0.94	0.47	0.48	0.51	0.55	0.60	0.181	0.255	0.227	0.786	0.548
Acetic	19.33	23.10	16.39	21.55	20.60	19.68	2.129	0.297	0.044	0.164	0.249
Propionic	7.35	9.27	7.36	10.01	8.92	9.78	0.649	0.562	0.001	0.136	0.570
iso-Butyric	1.23	1.29	1.30	1.23	1.41	1.27	0.099	0.913	0.972	0.439	0.924
Butyric	2.98	4.70	3.45	4.81	4.01	4.88	0.408	0.481	0.001	0.354	0.771
iso-Valeric	1.15	1.11	1.27	1.08	1.35	1.11	0.095	0.630	0.239	0.566	0.973
Valeric	1.10	1.38	1.30	1.26	1.50	1.34	0.098	0.647	0.233	0.167	0.765
Caproic	0.26	0.33	0.26	0.15	0.44	0.29	0.062	0.170	0.773	0.059	0.683
Total VFA	33.46	38.29	31.40	37.41	38.37	38.26	2.271	0.529	0.027	0.056	0.976
VFA, % of total^6^											
Acetic	56.97	55.08	51.84	53.48	53.49	51.18	1.845	0.075	0.946	0.531	0.136
Propionic	22.10	23.03	23.67	25.12	23.28	25.49	0.956	0.062	0.227	0.778	0.085
iso-Butyric	3.81	3.31	4.22	3.11	3.82	3.40	0.319	0.738	0.017	0.349	0.822
Butyric	9.22	11.45	11.08	11.89	10.59	12.68	0.754	0.136	0.053	0.671	0.291
iso-Valeric	3.63	2.89	4.13	2.76	3.67	2.99	0.330	0.570	0.003	0.306	0.819
Valeric	3.41	3.44	4.20	3.24	4.00	3.57	0.253	0.259	0.075	0.599	0.748
Caproic	0.87	0.80	0.87	0.41	1.15	0.68	0.185	0.294	0.175	0.283	0.642

^1^A BCVFA blend (isobutyrate, isovalerate, and 2-methyl butyrate – 1:1:1) was supplemented at 1% of the diet. BCVFA, branched-chain volatile fatty acids; NP, normal protein; LP, low protein; LF, low fiber; HF, high fiber; VFA, volatile fatty acid.

^2^Two additional diets without BCVFA were included (LP-LF and NP-HF).

^3^There was no interaction (*p* > 0.05) between fiber and protein levels on the ileal VFA concentration.

^4^Contrast: BCVFA supplemented diet vs. not supplemented diet.

^5^VFA, mmol/L: average millimoles of VFAs are present per liter of sample.

^6^VFA, % of total: Average of the percentage distribution of each VFAs within the samples of each diet.

The supplementation of BCVFA in NP-HF diet did not influence (*p* > 0.05) VFA concentration in ileal and fecal samples when compared to the non-supplemented NP-HF diet, except for isobutyric acid in ileal and proprionic acid in fecal samples from NP-fed pigs. The concentration (mmol/L and % of total) isobutyric acid in ileal samples from BCVFA supplemented pigs tended to be greater (*p* < 0.10) and propionic acid in fecal samples from unsupplemented pigs tended to be lower (*p* < 0.10). However, in LP-LF diet, lower concentrations (*p* < 0.1) of lactic, acetic, butyric, and total VFA production in ileal digesta were observed when BCVFA were supplemented. In fecal samples, caproic and total VFA production was decreased when BCVFA were supplemented in LP-LF diet.

### 3.3 Bacterial composition analysis

A comparative analysis of bacterial composition was conducted to identify gut microbial species that may respond to supplementation with isoacids. A total of 2.99 × 10^6^ high quality sequence reads, with a range of 12,779–57,981 reads/sample, were used to determine the taxonomic and OTU profiles of fecal bacterial communities in each treatment group. At the phylum level, *Bacillota* (formerly known as Firmicutes) were the most highly represented taxon, with an average of 74.2% across all samples (Table 9). Within this phylum, eight families (*Ruminococcaceae, Peptostreptococcaceae, Lactobacillaceae, Lachnospiraceae, Erysipelotrichaceae, Clostridiaceae 1, Clostridiales Incertae Sedis XIII,* and *Streptococcaceae*) were identified as most abundant. The second most highly represented phylum consisted of *Bacteroidota* (formerly known as Bacteroidetes), with an average of 9.8% across all samples; *Prevotellaceae* were identified as the most abundant family within this phylum. Other minor or lower abundance phyla were also identified, such as *Planctomycetes* and *Actinomycetota* (formerly known as Actinobacteria), which had an average abundance of 4.7 and 0.9%, respectively. Of particular interest in the context of this study on BCVFA supplementation, *Erysipelotrichaceae* were found to be more abundant in fecal communities from pigs fed NP-HF diets supplemented with BCVFA when compared to non-supplemented NP-HF diets (*p* < 0.05; Table 9).

**Table 9:** Effects of dietary treatment on the bacterial composition of fecal bacterial communities from growing pigs fed control or BCVFA-supplemented diets.

Taxa	BCVFA^1^				LP-LF	NP-HF	*p*–value^2^
	NP		LP				
	LF	HF	LF	HF			
Bacillota	72.33	74.44	77.77	76.18	78.92	65.74	0.056
Ruminococcaceae	14.48^ab^	17.27^a^	11.21^b^	14.01^ab^	12.65^ab^	18.14^a^	0.010
Clostridiaceae 1	16.65^abc^	9.02^c^	18.08^ab^	12.08^bc^	19.91^a^	7.70^c^	0.001
Peptostreptococcaceae	15.78	13.25	15.36	14.43	12.44	7.37	0.202
Lactobacillaceae	6.74	13.01	4.68	9.77	9.36	12.16	0.066
Lachnospiraceae	4.75^c^	8.15 ^ab^	4.32^c^	8.53^ab^	5.63^bc^	9.57^a^	0.001
Erysipelotrichaceae	5.77^bc^	3.91^c^	17.78^a^	8.29^bc^	10.94^ab^	1.40^d^	0.001
C. Incertae Sedis XIII	0.96	1.49	1.27	1.41	1.22	1.70	0.087
Streptococcaceae	1.27	0.78	0.53	1.12	1.74	0.58	0.526
Other Bacillota ^#^	5.93	7.55	4.54	6.54	5.04	7.13	-
Bacteroidota	8.37^b^	12.12^ab^	7.47^b^	9.48^ab^	9.01^ab^	12.49^a^	0.010
Prevotellaceae	5.22^abc^	8.18^a^	2.98^c^	5.25^abc^	4.91^bc^	7.85^ab^	0.002
Other Bacteroidota ^#^	3.16	3.94	4.49	4.23	4.10	4.63	-
Planctomycetes	6.17	3.44	4.83	3.84	2.78	7.05	0.053
Actinomycetota	0.52	1.26	1.00	0.79	1.13	0.70	0.179
Other phyla ^#&^	1.81	1.76	1.67	1.93	1.59	2.04	-
Unclassified Bacteria ^#^	10.38	6.57	6.72	7.37	6.17	11.62	-

Composition is shown by phylum and family using mean relative abundance (%).

^1^A BCVFA blend (isobutyrate, isovalerate, and 2-methyl butyrate – 1:1:1) was supplemented at 1% of the diet. BCVFA, branched-chain volatile fatty acids; NP, normal protein; LP, low protein; LF, low fiber; HF, high fiber.

^2^Superscripts ^abcd^ indicate significant difference at *p* ≤ 0.05.

^#^Statistical test not performed because of group heterogeneity.

^&^Group includes ten phyla with an average abundance < 1% across all samples.

To gain more insight on candidate bacterial species that may potentially be modulated by BCVFA supplementation, an analysis of bacterial composition at the OTU level was also performed. From a combined total of 18,376 OTUs identified across all samples, sixteen OTUs with an in-group average abundance of at least 1% in at least one dietary group were further analyzed (Table 10). As their respective sequence identities to the 16S rRNA gene of their respective closest valid relative were ≥ 97%, six of the most abundant OTUs were considered strains of characterized bacterial species, while the remaining ten OTUs likely corresponded to uncultured bacteria since they were too distant from any valid bacterial species. Two of the abundant OTUs identified as strains of known species, Ssd-23 and Ssd-110, were found to be more highly represented in the fecal bacterial communities of pigs fed the NP-HF diet when supplemented with BCVFA compared to the group fed the non-supplemented NP-HF diet (*p* < 0.05; Table 10). Similarly, OTU Ssd-1179 was in higher abundance in pigs fed the BCVFA-supplemented LP-LF diet compared to the group fed the non-supplemented diet LP-LF diet (*p* < 0.05). Notably, the abundance of OTU Ssd-1606 was lower in non-supplemented diets for both NP-HF and LP-LF conditions (*p* < 0.05).

**Table 10:** Effects of dietary treatment on the mean relative abundance (%) of the most abundant OTUs in fecal bacterial communities from growing pigs fed control or BCVFA-supplemented diets.

OTU	BCVFA^1^				LP-LF	NP-HF	Closest taxon (%id)	*p*-value^2^
	NP		LP					
	LF	HF	LF	HF				
Ssd-00110	4.80^bc^	2.44^c^	16.46^a^	7.13^b^	9.56^ab^	0.65^d^	*Turicibacter sanguinis* (97.0%)	0.001
Ssd-00134	13.81^ab^	5.69^c^	14.68^a^	7.44^bc^	14.86^a^	3.98^c^	*Cl. saccharoperbutylacetonicum* (97.0%)	0.001
Ssd-00014	12.83	10.79	9.92	9.75	7.60	6.53	*Ter. mayombei* (97.3%)	0.419
Ssd-00001	5.93^ab^	12.39^a^	4.28^b^	9.10^ab^	8.36^ab^	11.63^a^	*Lac. amylovorus* (99.6%)	0.041
Ssd-01095	6.06	3.38	4.74	3.76	2.72	6.88	*Lignipirellula cremea* (81.3%)	0.054
Ssd-01400	1.75	0.32	0.43	0.52	1.38	1.69	*Chr. massiliensis* (85.9%)	0.836
Ssd-00675	0.79	0.69	0.94	1.08	0.38	1.86	*Chr. massiliensis* (85.7%)	0.054
Ssd-00039	1.08	0.70	0.39	0.98	1.55	0.39	*Str. alactolyticus* (99.6%)	0.425
Ssd-01149	0.12	0.12	0.57	1.14	0.80	0.91	*Cop. phoceensis* (91.3%)	0.752
JPZ3-21748	0.32	0.88	0.84	1.57	0.78	2.60	*Eub. ruminantium* (91.1%)	0.255
Ssd-01079	3.02	0.96	1.61	1.34	0.82	2.24	*Mal. massiliense* (84.3%)	0.076
Ssd-00023	2.32^bc^	1.82^c^	4.60^a^	3.61^b^	4.01^ab^	0.51^d^	*Rom. timonensis* (98.4%)	0.001
Ssd-01606	0.09^ab^	0.02^bc^	0.01^c^	0.01^c^	0.32^b^	1.09^a^	*Cl. baratii* (96.8%)	0.001
Ssd-01179	0.11^c^	0.11^c^	1.27^a^	0.23^bc^	0.60^b^	0.06^c^	*Med. massiliensis* (90.0%)	0.001
Ssd-00936	0.92^ab^	1.11^a^	0.08^c^	0.48^bc^	0.53^bc^	0.93^ab^	*Duncaniella muris* (86.3%)	0.014
Ssd-00206	1.36	1.59	0.71	1.04	1.09	0.93	*Leyella stercorea* (89.6%)	0.050

^1^A BCVFA blend (isobutyrate, isovalerate, and 2-methyl butyrate – 1:1:1) was supplemented at 1% of the diet. BCVFA, branched-chain volatile fatty acids; NP, normal protein; LP, low protein; LF, low fiber; HF, high fiber.

^2^Superscripts ^abcd^ indicate significant differenCE at *p* ≤ 0.05.

Chr., Christensenella; Cl., Clostridium; Cop., Coprococcus; Eub., Eubacterium; Lac., Lactobacillus; Mal., Maliibacterium; Med., Mediterranea; Rom., Romboutsia; Str., Streptococcus; Ter.: Terrisporobacter.

## 4 Discussion

This experiment investigated the effects of dietary protein and fiber levels on nutrient digestibility, microbiota profiling, as well as fermentation products in BCVFA-fed pigs. To reduce the impact of each individual pig on the determined nutrient digestibility, a model that included the same pig to receive multiple experimental diets through the different collection periods was proposed. This helps to reduce animal-to-animal variation and allows the use of fewer animals making most judicious use of surgically modified pigs. Under this objective and experimental design, growth performance is not considered a good representation of the nutritional value of dietary treatments taking into consideration the short feeding period, the use of canulated pigs for the collection of digesta, and the potential confounding factor of the previous diet, which can be considered a limitation of this study. The microbial analysis was performed on fecal samples, which is not always a direct representation of small intestine microbiome where we found the most relevant responses in terms of digestibility (SID). This collection was not performed at the ileal level because microbiota composition in digesta samples can be easily altered by external factors associated with the canulated animal model (i.e., the presence of oxygen). However, the methods applied in this work are consistent with standard procedures to evaluate small intestinal nutrient digestibility in swine (41).

In this experiment, DDGS was used as a source of fiber in growing pig diets (HF = 16% and LF = 3%); this feed ingredient can be included up to 30% without altering development and nutrient digestibility (45). The inclusion levels were kept below this maximum threshold in order to determine the effect of BCVFA inclusion and their possible interaction with protein and fiber level. It was determined that the effect of BCVFA inclusion was dependent on fiber and protein content, given that AID and ATTD in each nutrient were different across diets.

Pigs fed BCVFA-supplemented NP-HF diet had the greatest AID of dietary fiber components and the greatest ATTD for ADF. This supports previous work in swine (26), where BCVFA supplementation at 1% increased apparent ileal digestibility and apparent total tract digestibility of crude fiber and hemicellulose in growing pigs fed corn-soybean meal diets. In addition, these results agree with the data published in ruminants that indicated an increase in fiber degradability when BCVFA are included in the diet (25). Because animal genomes do not encode enzymes that can effectively digest components of the plant cell wall, animal hosts are dependent on symbiotic gut microbial communities to metabolize plant fiber. It has been shown in ruminants that the inclusion of a blend of BCVFA increased fiber digestibility and altered ruminal bacterial diversity, particularly by promoting fiber-degrading bacteria (24, 46, 47). In cattle, a study showed a linear increase in ruminal bacteria in steers with increasing isovalerate supplementation (48). Similarly, Wang et.al (24) investigated the effect of different levels of iso-butyrate in the diet of cannulated steers and found that increasing supplemented levels of iso-butyrate resulted in a linear increase in cellulolytic bacterial species (*Ruminococcus albus, Ruminococcus flavefaciens, Butyrivibrio fibrisolvens* and *Fibrobacter succinogenes*) while protozoa and methanogen populations decreased. The present experiment agrees with the result previously mentioned in cattle because an increase was also evident in some families and more specifically in the abundance of certain OTUs.

Liu et al. (48) reported that isovalerate supplementation in steers improved rumen and total tract digestibility of DM and ADF and increased concentration of VFA and acetic, attributing these results to an improvement in ruminal degradation. However, the present experiment did not detect a relationship between digestibility and VFA concentration. The lower fermentation capacity in pigs compared to cattle may in part explain the lack of effect of BCVFA supplementation on fecal VFA content. In contrast, the AID of AA increased in pigs fed LP-LF control diet compared to BCVFA-supplemented LP-LF diet. The absence of BCVFA effect in LP-LF diets could be attributed to a lack of nitrogen availability for cellulolytic bacteria.

Another notable effect related to BCVFA supplementation was the increase in AID and ATTD of crude protein in diets with NP content compared with LP and non-supplemented diets. Although the concentration of BCVFA in ileal and feces depends on the inclusion level, source, and protein digestibility, studies in different species (human and rats) indicated that a high concentration of BCVFA in feces is related to a high protein content in diets (49). However, in this experiment, despite having an extra source of BCVFA, the concentration of iso-butyrate in feces did not differ between diets (supplemented vs. non-supplemented), but there was a higher concentration of isovalerate in the samples from pigs fed BCVFA-supplemented LP-LF diet. There is a scarcity of information regarding BCVFA, so further research is required to understand their metabolic pathways and their relationship with bacteria in the gastrointestinal tract. In ruminants, when ammonia is limiting BCVFA are more likely to be absorbed by the rumen epithelium where iso-valerate is metabolized in the rumen epithelium and iso-butyrate and 2-methyl butyrate are metabolized in the liver. A similar phenomena may be present in pigs with BCVFA supplementation in the presence of limited dietary protein (50) and may explain the lack of change in fecal branched-chain fatty acid concentrations.

Three OTUs, which were interpreted as corresponding to candidate fecal bacterial species or strains, were found to differ in abundance when comparing supplemented and non-supplemented treatment groups. While we were unable to gain further insight on the metabolic activities of OTU Ssd-1606 because it was too distant from its closest valid relative, OTUs Ssd-110 and Ssd-23 were identified as candidate strains of known or characterized species, which allowed more reliable predictions. Ssd-110 was in higher abundance in LF diets compared to HF diets, indicating that this OTU may be less competitive under conditions when fiber is included at higher levels in swine diets. Similarly, Ssd-110 was also more highly represented under conditions of low dietary proteins compared to normal protein levels. Supplementation with BCVFA appeared to relieve the apparent negative effects of higher fiber inclusion and/or protein levels on Ssd-110, with average abundance levels that were 3.75X greater in fecal communities from the BCVFA supplemented NP-HF diet compared to the non-supplemented control (Table 10). Ssd-110 was found to be most closely related to *Turicibacter sanguinis* (51); consistent with the results from this study, *Turicibacter* species have been reported as common residents of the gut environment in humans and animals (52–56). Ssd-110 was the only abundant OTU affiliated to the family *Erysipelotrichaceae*; on average, it represented 46.4–92.6% of sequences belonging to this family across all dietary treatments. From an in-house gene annotation analysis of a representative genome from *T. sanguinis* (Supplementary Table 1), coding sequences for a branched-chain amino acid aminotransferase (EC 2.6.1.42), as well as for a Na-dependent branched-chain amino acid transporter, were identified; the respective predicted functions of these bacterial proteins are consistent with the higher abundance of Ssd-110 that was observed when isoacid-supplemented diets were fed. While genes encoding for cellulases or for hemicellulose-hydrolyzing enzymes were not identified in the *T. sanguinis* genome analyzed, coding sequences for alpha-amylase were found; this supported the hypothesis that Ssd-110 would be more competitive when starch-rich ingredients are fed, compared to when diets with higher inclusion of fiber are used. Ssd-23 abundance across dietary treatments showed a pattern that was similar to the composition of Ssd-110. Gene annotation analysis of a representative genome from *Romboutsia timonensis*, the closest valid relative to Ssd-23, also revealed cellular functions consistent with isoacids supplementation, such as a branched-chain acyl kinase, a Na-dependent branched-chain amino acid transporters, as well as a branched-chain phosphotransacylases (Supplementary Table 2). Notably, BCFAs are predominant in cellular membranes of Gram-positive bacterial species such as *T. sanguinis* and *R. timonensis*; by regulating the types and abundance of BCFAs that are incorporated in their fatty acids, Gram-positive bacteria can adjust the biophysical properties of their cell membranes in response to changes in ambient conditions, such as temperature, pH or salinity (57).

## 5 Conclusion

Overall, 1% BCVFA inclusion in swine diets containing 17% neutral detergent fiber and 19% crude protein can optimize digestive efficiency, particularly at the ileal level, and can be potentially associated with microbiome modulation. The efficiency of BCVFA to improve nutrient digestibility can be impacted by the dietary protein level, with larger effects observed when normal crude protein levels were maintained in the diet, which can be related to the availability of nitrogen for the proliferation of cellulolytic bacteria. Finally, further research is needed to better understand the mechanism of action of BCVFA, along with more detailed investigation of the OTUs found to determine possible pathways in which BCVFA might be involved. It is also important to investigate and validate wherever the observed improvement in nutrients digestion translates in measurable performance responses in a full feeding trial focused in pig growth.

## Data Availability

Raw sequence data are available from the NCBI Sequence Read Archive under BioProject PRJNA1378998.

## References

[1] United Nations, Department of Economic and Social Affairs. World population prospects 2024: Ten key messages. (2024). Available online at: https://population.un.org/wpp/Publications/Files/WPP2024_Key-Messages.pdf [accessed 28 November 2024]

[2] Muscat A De Olde EM De Boer IJM Ripoll-Bosch R. The battle for biomass: a systematic review of food-feed-fuel competition. Glob Food Secur. (2020) 25:100330. doi: 10.1016/j.gfs.2019.100330

[3] Woyengo TA Beltranena E Zijlstra RT. Nonruminant nutrition symposium: controlling feed cost by including alternative ingredients into pig diets: a review. J Anim Sci. (2014) 92:1293–305. doi: 10.2527/jas.2013-7169, 24492540

[4] Urriola PE Cervantes-Pahm SK Stein HH. Fiber in swine nutrition In: Chiba LI, editor. Sustain. Swine Nutr. John Wiley & Sons Ltd., Indianapolis, IN. (2013). 255–76.

[5] Molist F Van Oostrum M Pérez JF Mateos GG Nyachoti CM Van Der Aar PJ. Relevance of functional properties of dietary fibre in diets for weanling pigs. Anim Feed Sci Technol. (2014) 189:1–10. doi: 10.1016/j.anifeedsci.2013.12.013

[6] Agyekum AK Nyachoti CM. Nutritional and metabolic consequences of feeding high-fiber diets to swine: a review. Engineering. (2017) 3:716–25. doi: 10.1016/J.ENG.2017.03.010

[7] Wenk C. The role of dietary fibre in the digestive physiology of the pig. Anim Feed Sci Technol. (2007) 90:21–33. doi: 10.1016/S0377-8401(01)00194-8

[8] Hung YT Zhu J Shurson GC Urriola PE Saqui-Salces M. Decreased nutrient digestibility due to viscosity is independent of the amount of dietary fibre fed to growing pigs. Br J Nutr. (2022) 127:177–87. doi: 10.1017/S0007114521000866, 33706826 PMC8756099

[9] Zijlstra RT Beltranena E. Feeding coproducts to pigs to reduce feed cost and reach sustainable food production. Anim Front. (2022) 12:18–22. doi: 10.1093/af/vfac067, 36530510 PMC9749814

[10] Stas EB DeRouchey JM Goodband RD Tokach MD Woodworth JC Gebhardt JT. Nutritional guide to feeding wheat and wheat co-products to swine: a review. Translational Anim Sci. (2024) 8:txae106. doi: 10.1093/tas/txae106 PMC11439155 39346699

[11] Aderibigbe AS Park CS Johnson T Velayudhan DE Vinyeta E Adeola O. Efficacy of a novel multi-enzyme feed additive on growth performance, nutrient digestibility, and gut microbiome of weanling pigs fed corn–wheat or wheat–barley-based diet. J of Anim Sci. (2024) 102:skae064. doi: 10.1093/jas/skae064 38466229 PMC10977034

[12] Liu Y Espinosa CD Abelilla JJ Casas GA Lagos LV Lee SA . Non-antibiotic feed additives in diets for pigs: a review. Anim Nutr. (2018) 4:113–25. doi: 10.1016/j.aninu.2018.01.007, 30140751 PMC6103469

[13] Barbosa KA Genova JL Pazdziora ML de Azevedo LB Wendt GN Rupolo PE . Effects of combined feed additives in diets to support growth performance and intestinal health profile in nursery piglets. Livest Sci. (2022) 266:105121. doi: 10.1016/j.livsci.2022.105121

[14] Liu Q Wang C Huang YX Dong KH Yang WZ Zhang SL . Effects of isovalerate on ruminal fermentation, urinary excretion of purine derivatives and digestibility in steers. J Anim Physiol Anim Nutr. (2009) 93:716–25. doi: 10.1111/j.1439-0396.2008.00861.x, 19138353

[15] Liu Q Wang C Zhang YL Pei CX Zhang SL Wang YX . Effects of isovalerate supplementation on growth performance and ruminal fermentation in pre- and post-weaning dairy calves. J Agric Sci. (2016) 154:1499–508. doi: 10.1017/S0021859616000630

[16] Salazar N González S de los Reyes Gavilan CG Rios-Covian D. (2022). Branched short-chain fatty acids as biological indicators of microbiota health and links with anthropometry. In: VB, Patel and VR Preedy, editors. Biomarkers in Nutrition. Biomarkers in Disease: Methods, Discoveries and Applications. Springer, Cham. doi: 10.1007/978-3-030-81304-8_4-1

[17] Tardiolo G La Fauci D Riggio V Daghio M Di Salvo E Zumbo A . Gut microbiota of ruminants and monogastric livestock: an overview. Animals. (2025) 15:758. doi: 10.3390/ani15050758, 40076043 PMC11899476

[18] Dal Pont GC Eyng C Bortoluzzi C Kogut MH. Enzymes and gut health in Monogastric animals: Effects beyond digestibility, vol. 4. Cham: Springer (2022).

[19] Roman-Garcia Y Denton BL Mitchell KE Lee C Socha MT Firkins JL. Conditions stimulating neutral detergent fiber degradation by dosing branched-chain volatile fatty acids. I: comparison with branched-chain amino acids and forage source in ruminal batch cultures. J. Dairy Sci. (2021) 104:6739–55. doi: 10.3168/jds.2020-20054, 33814156

[20] Roman-Garcia Y Mitchell KE Denton BL Lee C Socha MT Wenner BA . Conditions stimulating neutral detergent fiber degradation by dosing branched-chain volatile fatty acids. II: relation with solid passage rate and pH on neutral detergent fiber degradation and microbial function in continuous culture. J Dairy Sci. (2021) 104:9853–67. doi: 10.3168/jds.2021-20335, 34147227

[21] Roman-Garcia Y Mitchell KE Lee C Socha MT Park T Wenner BA . Conditions stimulating neutral detergent fiber degradation by dosing branched-chain volatile fatty acids. III: relation with solid passage rate and pH on prokaryotic fatty acid profile and community in continuous culture. J Dairy Sci. (2021) 104:9868–85. doi: 10.3168/jds.2021-20336, 34253360

[22] Allison MJ Bryant MP. Biosynthesis of branched-chain amino acids from branched-chain fatty acids by rumen bacteria. Arch Biochem Biophys. (1963) 101:269–77. doi: 10.1016/S0003-9861(63)80012-0, 14012183

[23] Mitchell KE Kienzle SL Lee C Socha MT Kleinschmit DH Firkins JL. Supplementing branched-chain volatile fatty acids in dual-flow cultures varying in dietary forage and corn oil concentrations. II: biohydrogenation and incorporation into bacterial lipids. J Dairy Sci. (2023) 106:7548–65. doi: 10.3168/jds.2022-23192 37532628

[24] Wang C Liu Q Zhang YL Pei CX Zhang SL Wang YX . Effects of isobutyrate supplementation on ruminal microflora, rumen enzyme activities and methane emissions in Simmental steers. J Anim Physiol Anim Nutr. (2014) 99:123–31. doi: 10.1111/jpn.12191 24702602

[25] Copelin JE Firkins JL Socha MT Lee C. Effects of diet fermentability and supplementation of 2-hydroxy-4-(methylthio)-butanoic acid and isoacids on milk fat depression: 1. Production, milk fatty acid profile, and nutrient digestibility. J Dairy Sci. (2021) 104:1591–603. doi: 10.3168/jds.2020-18949, 33309372

[26] Benavides-Infante AP Rodrigues LA Socha MT Schweer WP Levesque CL Perez-Palencia JY. Effect of increasing dietary isoacid levels on total tract and apparent ileal nutrient digestibility and fermentation products in growing pigs fed corn-soybean meal diets. J Animal Sci Biotechnol. (2025) 16:102. doi: 10.1186/s40104-025-01239-0, 40676664 PMC12272956

[27] NRC. Nutrient requirements of swine: Eleventh revised edition. Washington, DC: The National Academies Press (2012).

[28] Zhang F Adeola O. Techniques for evaluating digestibility of energy, amino acids, phosphorus, and calcium in feed ingredients for pigs. Anim Nutr. (2017) 3:344–52. doi: 10.1016/j.aninu.2017.06.008, 29767105 PMC5941275

[29] Kim BG Lee SA Park KR Stein HH. At least 3 days of adaptation are required before indigestible markers (chromium, titanium, and acid insoluble ash) are stabilized in the ileal digesta of 60-kg pigs, but values for amino acid digestibility are affected by the marker. J Anim Sci. (2020) 98:skaa027. doi: 10.1093/jas/skaa027, 31999323 PMC7041900

[30] Sappok MA Perez Gutierrez O Smidt H Pellikaan WF Verstegen MWA Bosch G . Adaptation of faecal microbiota in sows after diet changes and consequences for in vitro fermentation capacity. Anim. (2015) 9:1453–64. doi: 10.1017/S1751731115000865, 25997358

[31] Myers WD Ludden PA Nayigihugu V Hess BW. Technical note: a procedure for the preparation and quantitative analysis of samples for titanium dioxide. J Anim Sci. (2004) 82:179–83. doi: 10.2527/2004.821179x, 14753360

[32] Bech N Jensen PA Dam-Johansen K. Determining the elemental composition of fuels by bomb calorimetry and the inverse correlation of HHV with elemental composition. Biomass Bioenergy. (2009) 33:534–7. doi: 10.1016/j.biombioe.2008.08.015.

[33] Darwin N WipaCharles N Cord-Ruwisch R. Concurrent lactic and volatile fatty acid analysis of microbial fermentation samples by gas chromatography with heat pre-treatment. J Chromatogr Sci. (2018) 56:1–5. doi: 10.1093/chromsci/bmx086, 29069353

[34] Izuddin WI Loh TC Samsudin AA Foo HL Humam AM Shazali N. Effects of postbiotic supplementation on growth performance, ruminal fermentation and microbial profile, blood metabolite and GHR, IGF-1 and MCT-1 gene expression in post-weaning lambs. BMC Vet Res. (2019) 15:315. doi: 10.1186/s12917-019-2064-9., 31477098 PMC6719353

[35] Novamsky I Van Eck R Van Schouwenburg C Walinga I. Total nitrogen determination in plant material by means of the indophenol-blue method. J Life Sci. (1974) 22:3–5. doi: 10.18174/njas.v22i1.17230

[36] Yu Z Morrison M. Improved extraction of PCR-quality community DNA from digesta and fecal samples. BioTechniques. (2004) 36:808–12. doi: 10.2144/04365ST04, 15152600

[37] Edwards U Rogall T Blöcker H Emde M Böttger EC. Isolation and direct complete nucleotide determination of entire genes. Characterization of a gene coding for 16S ribosomal RNA. Nucl Acids Res. (1989) 17:7843–53. doi: 10.1093/nar/17.19.7843, 2798131 PMC334891

[38] Opdahl LJ Gonda MG St Pierre B. Identification of uncultured bacterial species from Firmicutes, Bacteroidetes, and *CANDIDATUS* Saccharibacteria as candidate cellulose utilizers from the rumen of beef cows. Microorganisms. (2018). 6:17. doi: 10.3390/microorganisms6010017 29495256 PMC5874631

[39] Schloss PD Westcott SL Ryabin T Hall JR Hartmann M Hollister EB . Introducing mothur: open-source, platform-independent, community-supported software for describing and comparing microbial communities. Appl Environ Microbiol. (2009) 75:7537–41. doi: 10.1128/AEM.01541-09, 19801464 PMC2786419

[40] Wang Q Garrity GM Tiedje JM Cole JR. Naive Bayesian classifier for rapid assignment of rRNA sequences into the new bacterial taxonomy. App Environ Microbiol. (2007) 73:5261–7. doi: 10.1128/AEM.00062-07, 17586664 PMC1950982

[41] Altschul SF Madden TL Schäffer AA Zhang J Zhang Z Miller W. Gapped BLAST and PSI-BLAST: a new generation of protein database search programs. Nucleic Acids Res. (1997) 25:3389–402. doi: 10.1093/nar/25.17.3389, 9254694 PMC146917

[42] Stein HH Fuller MF Moughan PJ Sève B Mosenthin R Jansman AJM . Definition of apparent, true, and standardized ileal digestibility of amino acids in pigs. Livest Sci. (2007) 109:282–5. doi: 10.1016/j.livsci.2007.01.019

[43] Kong C Adeola O. Evaluation of amino acid and energy utilization in feedstuff for swine and poultry diets. Asian Australas J Anim Sci. (2014) 27:917–25. doi: 10.5713/ajas.2014.r.02, 25050031 PMC4093562

[44] Adeola O. Digestion and balance techniques in pigs Second. Boca Raton, FL: CRC Press eBooks 2001 903–916

[45] Stein HH Shurson GC. Board-invited review: the use and application of distillers dried grains with solubles in swine diets. J Anim Sci. (2009) 87:1292–303. doi: 10.2527/jas.2008-1290, 19028847

[46] Liu Q Wang C Huang Y Dong K Wang H Yang W. Effects of isobutyrate on rumen fermentation, urinary excretion of purine derivatives and digestibility in steers. Arch Anim Nutr. (2008) 62:377–88. doi: 10.1080/17450390802327761, 18942585

[47] Jiang F Gao Y Peng Z Ma X You Y Hu Z . Isoacids supplementation improves growth performance and feed fiber digestibility associated with ruminal bacterial community in yaks. Front Microbiol. (2023) 14:1175880. doi: 10.3389/fmicb.2023.1175880, 37396385 PMC10311502

[48] Liu Q Wang C Pei CX Li HY Wang YX Zhang SL . Effects of isovalerate supplementation on microbial status and rumen enzyme profile in steers fed on corn Stover based diet. Livest Sci. (2014) 161:60–8. doi: 10.1016/j.livsci.2013.12.034

[49] Bartlett A Kleiner M. Dietary protein and the intestinal microbiota: an understudied relationship. Sci. (2022) 11:105313. doi: 10.1016/j.isci.2022.105313, 36339270 PMC9626677

[50] Firkins JL Mitchell KE White AF. Invited review: role for isoacids in dairy nutrition. Appl Anim Sci. (2024) 40:466–77. doi: 10.15232/aas.2024-02537

[51] Bosshard PP Zbinden R Altwegg M. *Turicibacter sanguinis* gen. Nov., sp. nov., a novel anaerobic, gram-positive bacterium. Int J Syst Evol Microbiol. (2002) 52:1263–6. doi: 10.1099/00207713-52-4-1263. 12148638 doi: 10.1099/00207713-52-4-1263, 12148638

[52] Yano JM Yu K Donaldson GP Shastri GG Ann P Ma L . Indigenous bacteria from the gut microbiota regulate host serotonin biosynthesis. Cell. (2015) 161:264–76. doi: 10.1016/j.cell.2015.02.047, 25860609 PMC4393509

[53] Kim CY Lee M Yang S Kim K Yong D Kim HR . Human reference gut microbiome catalog including newly assembled genomes from under-represented Asian metagenomes. Genome Med. (2021) 13:134. doi: 10.1186/s13073-021-00950-7, 34446072 PMC8394144

[54] Huang S Ji S Yan H Hao Y Zhang J Wang Y . The day-to-day stability of the ruminal and fecal microbiota in lactating dairy cows. Microbiol Open. (2020). 9:e990. doi: 10.1002/mbo3.990, 32175695 PMC7221419

[55] Maki JJ Looft T. Turicibacter bilis sp. nov., a novel bacterium isolated from the chicken eggshell and swine ileum. Int J Syst Evol Microbiol. (2022) 72:005153. doi: 10.1099/ijsem.0.005153, 35084297 PMC8895650

[56] Feng Y Wang Y Zhu B Gao GF Guo Y Hu Y. Metagenome-assembled genomes and gene catalog from the chicken gut microbiome aid in deciphering antibiotic resistomes. Commun Biol. (2021) 4:1305. doi: 10.1038/s42003-021-02827-2, 34795385 PMC8602611

[57] Kaneda T. Iso- and anteiso-fatty acids in bacteria: biosynthesis, function, and taxonomic significance. Microbiol Rev. (1991) 55:288–302. doi: 10.1128/mr.55.2.288-302.1991, 1886522 PMC372815

[58] Kim M Morrison M Yu Z. (2011). Evaluation of different partial 16S rRNA gene sequence regions for phylogenetic analysis of microbiomes. J. Microbiol. Methods. 84:81–87. doi: 10.1016/j.mimet.2010.10.020, 21047533

[59] Johnson JS Spakowicz DJ Hong BY Petersen LM Demkowicz P Chen L . (2019). Evaluation of 16S rRNA gene sequencing for species and strain-level microbiome analysis. Nature communications, 10:5029. doi: 10.1038/s41467-019-13036-1, 31695033 PMC6834636

